# Microfluidic Analysis for the Determination of Protein Content in Different Types of Plant-Based Drinks

**DOI:** 10.3390/molecules28186684

**Published:** 2023-09-18

**Authors:** Fruzsina Balogh-Hartmann, Csilla Páger, Anita Bufa, Ibolya Madarászné Horváth, Zsófia Verzár, Tamás Marosvölgyi, Lilla Makszin

**Affiliations:** 1 Institute of Bioanalysis, Medical School, Szentágothai Research Center, University of Pécs, 7622 Pécs, Hungary; fruzsina.hartmann@aok.pte.hu (F.B.-H.); csilla.pager@aok.pte.hu (C.P.); anita.bufa@aok.pte.hu (A.B.); ibolya.madarasz@aok.pte.hu (I.M.H.); marosvolgyi.tamas@pte.hu (T.M.); 2 Institute of Nutritional Sciences and Dietetics, Faculty of Health Sciences, University of Pécs, 7621 Pécs, Hungary; verzar.zsofia@pte.hu

**Keywords:** plant-based milk alternatives, food allergy, protein profile, total protein content, microchip gel electrophoretic method

## Abstract

The widespread consumption of plant-based drinks, driven by health and dietary reasons (including cow’s milk allergy, lactose intolerance, milk protein intolerance, following a vegetarian or vegan diet) necessitates automated and accurate test methods. Our study demonstrates the simultaneous determination of protein components and total protein concentrations in plant-based milk alternatives using a rapid and reproducible microchip gel electrophoretic method. As expected, the electrophoretic profiles of each plant-based drink differed. Based on our analyses and statistical evaluation, it can be determined that the protein profiles of different plant-based beverages do not differ significantly between different manufacturers or different expiry dates. The measured total protein content was compared with the nominal values, i.e., the values stated on the beverage labels. As the number of consumers of functional and specialized plant-based milk alternatives continues to rise, it is important to prioritize methods that provide qualitative and quantitative information on protein composition and other nutrients.

## 1. Introduction

Nowadays, people avoid milk and dairy products due to lactose intolerance and milk protein allergies, and more and more people follow vegetarian and vegan diets. In addition, many people are concerned about the presence of antibiotic residues in dairy products for health reasons. While the demand for cow’s milk decreased by 7% [1], a 9% increase was observed in the demand for vegetable beverages in 2015. Consumer demand is shifting towards lactose-free products and plant-based beverages [2].

Cow’s milk allergy is not the same as lactose intolerance. Milk protein intolerance is mainly caused by the presence of beta-lactoglobulin, alpha-lactalbumin and bovine serum albumin, and it can often occur in early childhood due to the premature introduction of milk in the diet (before 12 months of age). In developed countries, its prevalence can be as high as 3%. Sensitivity to lactose is caused by the partial or complete lack of the lactase enzyme [3]. Accordingly, many people are forced to consume dairy alternatives, while others follow a vegan diet in line with gastronomic trends [4,5]. Although there are numerous innovative plant-based food and drink alternatives to cow’s milk, many of these foodstuffs face different technological problems such as processing, preservation, and seeking flavor and texture similarities. Most of the milk alternatives lack nutrient balance (compared to cow’s milk) but may contain functionally active ingredients that are attractive to health-conscious consumers because of their health-promoting properties [5,6].

In accordance with the EU Council Regulation and Directive 90/496/EEC of 2011, the values on food labels, including different brands of milk on the market, are average values that best represent the actual energy and nutrient values [7]. The Allergen Nomenclature is an official site approved by the World Health Organization (WHO) and the International Union of Immunological Societies (IUIS) [8].

PBMAs are classified into five different categories [6], out of which we have dealt with representatives from the following three categories of plant-based beverages: cereal-based (oat drink, rice drink), legume-based (soy drink, chickpea drink), and nut-based (almond drink, coconut drink, cashew drink).

*Oats*, which are used to make cereal-based plant drinks, have unique characteristics compared to other cereals when it comes to their protein composition, concentration, and stability. Proteins in oats, along with β-glucan, can form aggregates during complexation, which is an important property [9]. During an analysis of oat proteins using size exclusion chromatography, the protein solution was first dispersed with 0.2 M NaCl and 0.2 M sodium phosphate buffer and then cooled. The samples were centrifuged at 10,000 rpm for 10 min at 10 °C. This separation method revealed the presence of several protein fractions in oats, with the major constituent being the 12S-globulin protein fraction. The 12S-globulin was found in hexametric form and accompanied by 7S-globulins, 3S-globulins, albumin, and prolamins [10]. Apart from being a source of protein, oats are also utilized as a plant-based gelling agent. This property makes oats versatile and suitable for various food applications [9,10].

Another important example of cereal-based plant drinks is rice drink. In an analysis of rice bran proteins, the albumin and globulin fractions were isolated and subjected to sodium dodecyl sulfate–polyacrylamide gel electrophoresis (SDS-PAGE) under reducing conditions. For the globulin fraction, the main band appeared between 13 and 28 kDa, with additional bands observed at 40, 36, and 22 kDa. In the albumin fraction, the dominant band appeared at 13 kDa, along with bands at 38, 33, and 24 kDa. When the fractions were hydrolyzed, the gel appearance changed [11]. Another study focused on four rice protein fractions, namely glutelin, globulin, albumin, and prolamine, which were tested after heat treatment under reducing and non-reducing conditions. The results indicated that there were no significant changes in the reduced state after heat treatment [12]. In terms of allergenicity, an SDS-PAGE analysis identified three determinant bands of potential rice allergens at 9, 14, and 31 kDa. The official allergen database includes Ory s 1 and Ory s 12, which are related to pollen allergens [8]. Rice consumption is indeed recommended for individuals with cereal allergies as it is considered to be a non-allergenic grain [13]. Rice is widely consumed worldwide, and its popularity as a staple food is reflected in global consumption figures. According to the forecast of the Food and Agriculture Organization (FAO) for 2018, world rice consumption was projected to reach 509.1 million tons [14]. Rice is a key food source for many regions and plays a significant role in global food security. 

As for leguminous PBMAs, their sensory properties are the main limiting factors of their widespread uptake [6]. Soy- and chickpea-based plant drinks have attracted our focus in this category.

*Soybeans* are known for their high protein content, and more than 16 types of proteins have been identified in soybeans. The majority of these proteins, approximately 70–80%, belong to the globulin fraction, while the remaining proteins are classified as albumins. An SDS-PAGE analysis has revealed that the globulin fraction of soybeans consists of two major proteins: β-conglycine and glycine. β-conglycine is a trimer with a molecular weight of approximately 180 kDa, and it is composed of three major subunits, α’, α, and β subunits, with molecular weights of approximately 76, 72, and 53 kDa, respectively. Glycine, on the other hand, has a molecular weight of around 320 kDa and is composed of six units. Each unit consists of an acidic and a basic polypeptide subunit linked by disulfide bridges. The acidic subunit has a molecular mass of 34–45 kDa, while the basic subunit is approximately 19–22 kDa [15,16]. In addition to β-conglycine and glycine, smaller amounts of other protein components have been identified in soybeans. These include agglutinin (34–36 kDa), β-amylase (55 kDa), and Kunitz trypsin inhibitor (21 kDa). Furthermore, an additional band at 23 kDa has been observed in the globulin fraction [17]. Soy is considered a common allergen source, necessitating the development of analytical methods that can accurately detect and quantify soy proteins and their allergenic components [16].

*Chickpeas* are known for their nutritional value and have been found to contain important components that may contribute to disease prevention. These include isoflavones, polyunsaturated fatty acids, and minerals which have been studied for their potential health benefits. Incorporating chickpeas into a healthy diet can provide these beneficial components [18]. In terms of their protein composition, chickpea proteins are primarily composed of globulins, with the major globulin known as 11S legumin. Other protein fractions found in chickpeas include glutelins, albumins, and prolamins [19]. Protein fraction analysis using capillary electrophoresis under reducing and non-reducing conditions has provided insights into the composition of these proteins. Under reducing conditions, both β- and α-subunits of legumin can be observed on electropherograms, with the former appearing at molecular weights of 35–40 kDa and the latter at 20 kDa. Under non-reducing conditions, in addition to the major fractions, the presence of 7S vicilin has been identified, with a major band at 50 kDa and minor bands at 15, 32, and 70 kDa. Other protein components, such as glutelin (20–55 kDa), convicilin (~70 kDa), and lipoxygenase enzyme (92–94 kDa), have also been detected [19]. Chickpeas have been considered as a potential alternative to soybean, as both legumes are rich in fiber, protein, minerals, and isoflavones. These characteristics make them suitable for their inclusion in a healthy diet [18,20].

The third category of PBMAs we analyzed includes nut-based plant drinks, namely almond-, coconut- and cashew-based plant drinks.

*Almonds* are widely consumed worldwide and can be enjoyed in various forms, either raw or processed. They are commonly used in confectionery, but they are also used to produce almond drinks, providing a plant-based alternative to dairy milk. Due to the prevalence of almond allergies, it is a priority for the food industry to prevent cross-contamination with almond-containing products [21]. Almond allergens have been identified and listed in the Allergen Nomenclature. There are seven known allergens associated with almonds. The proteins in almonds, specifically in the prunin fraction, have been analyzed using SDS-PAGE. The two major proteins in the prunin fraction are prunin-1 (Pru-1) and prunin-2 (Pru-2), with approximate molecular weights of 61 and 63 kDa, respectively. These proteins can be further broken down into acidic and basic subunits through the action of DTT (dithiothreitol). The acidic subunit of Pru-1 weights 40.1 kDa, while the basic subunit weights 20.9 kDa. The acidic subunit of Pru-2 weights 34.5 kDa, and the basic subunit weights 21.4 kDa. These two subunits combine to form amandin, that is not only the major protein fraction, but also the primary allergen present in almonds [21,22]. Additionally, other allergenic proteins have been identified in almonds, including Pru du 4, with a molecular weight of 14 kDa, and Pru du 5, with a molecular weight of 11 kDa [21]. While almonds are considered a healthy food, individuals with even a minimal sensitivity to the allergens contained in almonds can experience mild to severe allergic symptoms [21]. It is important for individuals with almond allergies to be cautious and avoid almond-containing products to prevent allergic reactions. Furthermore, food manufacturers strive to maintain strict allergen control measures to minimize the risk of cross-contamination and ensure the safety of consumers with almond allergies.

*Coconut* drink is produced from coconut pulp, and it is an oil-in-water emulsion. The stability of coconut drink is attributed to the proteins it contains [23]. The protein content of coconut meat typically ranges from 2.6% to 5.6%. Five protein fractions have been identified and isolated from defatted coconut flour: albumin (21% *w*/*w*), globulins (40% *w*/*w*), prolamins (3.3% *w*/*w*), glutelin-1 (14.4% *w*/*w*), and glutelin-2 (4.8% *w*/*w*). Various methods, such as SDS-PAGE, gel filtration, differential scanning calorimetry (DSC), and amino acid analysis have been used for their isolation and characterization [24]. After their extraction from coconut using 0.4 M NaCl buffer, the presence of globulins has been determined by gel filtration. Globulins, specifically the 11S globulin known as cocosin, constitute a major portion of coconut proteins. Cocosin has a molecular weight of 326 kDa and represents around 86% (*w*/*w*) of the total globulin content [25]. Another globulin, 7S globulin, has a molecular weight of 156 kDa and accounts for 14% (*w*/*w*) of the globulin content. SDS-PAGE analysis has revealed that cocosin exhibits two closely migrating bands at 34 kDa (acidic polypeptide) and 24 kDa (basic polypeptide), while 7S globulin exhibits three bands at 16, 22, and 24 kDa [25]. Under reducing conditions, the protein pattern of cocosin consists of bands at 55, 46, 33, 25, 18, and 16 kDa [23]. Regarding allergens in coconuts, currently only one allergen, Coc n 1, belonging to the vicilin family of proteins, has been officially accepted. However, a study conducted in the United States suggested that the risk of coconut allergy in children who are allergic to peanuts and tree nuts is not significant [26]. Coconut drink not only serves as a milk alternative, but it is also used as an ingredient in various food products. It is rich in nutrients, vitamins (such as vitamin E and A), and minerals (including iron, magnesium, calcium, potassium, and zinc). Despite its positive properties, coconut consumption is limited due to its high saturated fatty acid content [6]. The protein fractions present in coconut milk can be utilized as natural antioxidants or to extend the shelf life of food products [27].

*Cashew nuts* are known for their beneficial effects, including a lower risk of cardiovascular disease and other health benefits. The proteins present in cashew nuts have been studied under different conditions to understand their characteristics. Under non-reducing conditions, five major polypeptides have been identified, with molecular weights of 6.5, 19, 34, 44, and 53 kDa. A SDS-PAGE separation of cashew protein components under reducing conditions resulted in a 53 kDa fraction with two bands at 21 and 32 kDa. Glutelin had a larger molecular weight [28]. While cashew nuts have the potential to serve as an excellent substitute in the food industry, it is crucial to consider their allergenic properties. Cashews are known to contain specific allergens, including Ana o 1, Ana o 2, and Ana o 3, which are listed in allergen databases. These allergenic proteins can trigger allergic reactions in individuals with cashew allergies. It is important for food manufacturers and individuals with allergies to be aware of these allergens to prevent adverse reactions. Cashew allergies can range from mild to severe, and individuals allergic to cashews must avoid consuming cashew-containing products. Cross-reactivity between cashews and other tree nuts, such as pistachios and mangoes, is also possible, so caution should be exercised when consuming or handling these foods.

Reducing allergenic reactions is an important consideration in vegetable drink production. Selecting plants with fewer allergens and implementing measures to monitor and control allergenic factors throughout the production process, including growing conditions, handling, storage, heat treatment, and enzymatic modifications, can help mitigate the risk of allergic reactions [15]. In the case of almonds, autoclave treatment has been explored as a method to reduce both proteins and allergens. While these studies have shown some reduction in allergenic content, further research and development are necessary to effectively eliminate the total allergenic content from almonds [21]. Food allergies can lead to a range of symptoms, from mild discomfort (coughing, itching, rash, vomiting, and diarrhea) to severe reactions like anaphylactic shock [29]. Analyzing protein fractions and total protein content in plant milk drinks is indeed crucial. This information helps to identify potential allergens and ensures that the beverages are safe for individuals with food allergies. By combining non-allergenic plant beverages, it is possible to create milk substitutes that have adequate protein profiles and can be consumed by those with specific dietary restrictions. Plant-based milk alternatives are increasingly popular, not only as substitutes for individuals with food allergies but also as more affordable options, particularly for lower-income groups in developing countries. In regions where the supply of cow’s milk may be insufficient or less accessible, plant-based milk alternatives provide a viable and cost-effective alternative [6]. Overall, the availability of non-allergenic plant beverages and an increasing awareness of food allergies contribute to the growing acceptance and consumption of PBMAs, benefiting individuals with dietary restrictions and populations in need of affordable dairy substitutes.

Direct and indirect methods are available for the determination of food proteins. The choice between direct and indirect methods depends on several factors, including the nature of the sample, the level of accuracy required, and the available equipment. Direct methods are often preferred when the specific protein of interest is known, while indirect methods can provide a total nitrogen estimate, which can be useful for samples with complex matrices. We have to note that each method has its own set of limitations and potential interferences, which should be considered before selecting the most appropriate method for a given determination to ensure accurate results. The Bradford method is a widely used technique for protein quantification due to its sensitivity and rapid method. However, the method may not be ideal for all types of proteins, especially if they have significantly different amino acid compositions or they are present in complex mixtures, like plant-based drink alternatives. While the Dumas and Kjeldahl methods are valuable for total nitrogen content determination in various samples, they may not always be the best choice for complex biological samples due to matrix effects and the need for extensive sample preparation [30].

Several studies have been conducted on the analytical methods for protein determination in plant-based milk alternatives (PBMAs) [6,17,23,28,31,32]. The available studies on protein determination in PBMAs are scarce, especially those using a low sample, high sensitivity, rapid and reproducible microfluidic method [33]. In contrast to SDS-PAGE, the microfluidic assay is suitable for the rapid qualitative and quantitative analysis of protein fractions in parallel, as well as for determining total protein content with high reproducibility.

The aim of this study was to optimize and apply the microchip gel electrophoretic (MGE) method in order to analyze protein fractions in plant-based drinks. We sought to obtain a comprehensive profile of the protein components present in these drinks. Additionally, our aim was to determine and compare the total protein content stated on the food labels of these plant-based drinks. Furthermore, the study intended to compare the protein content among products of the same type but from different brands and with varying expiry dates. This comparison will provide insights into potential variations of protein content within the same product category.

## 2. Results and Discussion

### 2.1. Evaluation of Total Protein Content

A total of 100 plant-based drinks were initially selected; however, only 15 products which did not contain any additives, with the exception of salt, were included in this particular study. The analysis of the other products will be presented in a separate work, with the aim of examining the impact of additives on both the protein profile and nitrogen content of plant-based drinks. This approach allows for a more focused and detailed analysis of the specific factors that may influence the nutritional content of these beverages. The current study analyzed a total of 15 plant-based drinks, including a variety of different types such as oat, rice, chickpea, soy, coconut, cashew and almond. Each of these drinks was tested with three different expiry dates, resulting in a total sample size of 45.

According to the EU Council Regulation no. 1169/2011 [7], it is mandatory for all plant-based drink types to report their nutritional information, including total protein content (g/100 mL). In this article, the total protein content of different PBMAs was analyzed.

The quantification is based on the percentage distribution of each protein fraction and the time-corrected peak area. These results were obtained by the manual integration of the peaks. Total protein content was calculated according to the original protocol using the equation below [34]. In determining the total protein content of plant-based drinks, the area and concentration below the time-corrected peak of the ladder were considered, as well as the dilutions used in sample preparation.

The evaluation of total protein concentrations (TPs) compared to nominal values is shown in Table 1.

Total protein content of the measured plant-based drinks was as follows: 2.0 (1.9–3.2) g/100 mL of oat drink, 0.35 (0.25–0.4) g/100 mL of rice drink, 2.6 (2.2–2.8) g/100 mL of chickpea drink, 3.8–4.7 (3.5–5.1) g/100 mL of soy drink, 2.0 (1.7–2.8) g/100 mL of coconut drink, 1.8 (1.75–1.85) g/100 mL of cashew drink, and 2.6 (1.9–2.8) g/100 mL of almond drink. The results showed that total protein content significantly differs among product types (*p* < 0.001). Soy drinks had the highest measured total protein content (4.7 g/100 mL), while rice drinks contained the lowest (0.35 g/100 mL). After multiple pairwise comparisons, some significant differences were observed. The rice–almond (*p* = 0.018), rice–oat (*p* = 0.049), rice–soy (*p* < 0.001), coconut–soy (*p* < 0.001), cashew–soy (*p* = 0.005), and almond–soy (*p* = 0.045) pairs showed significant differences. However, there was no difference in total protein content between brands of almond (*p* = 0.184), coconut (*p* = 0.436), rice (*p* = 0.157), and soy (*p* = 0.155) drinks. Based on the given information (nominal TP), the measured total protein content complies with the requirements and has a tolerance of ±2 g/100 g below 10 g/100 g. The microchip gel electrophoretic HSP 250 method can accept ±3% CV (coefficient of variation) for scale reproducibility in the molecular weight range 10–250 kDa and ±10% CV for sizing accuracy. For concentration determination, the peak area of the measured protein was considered, and the method allows a tolerance of ±20% CV for the reproducibility of the peak area. Statistically, the measured total protein content, when compared to the information on the label, differed significantly (*p* < 0.001). This indicates that the measured total protein content (measured TP) in these drinks may have been higher than what is stated on the label (nominal TP) (Table 1).

### 2.2. Protein Content of Plant-Based Milk Alternatives

**Figure 1:**
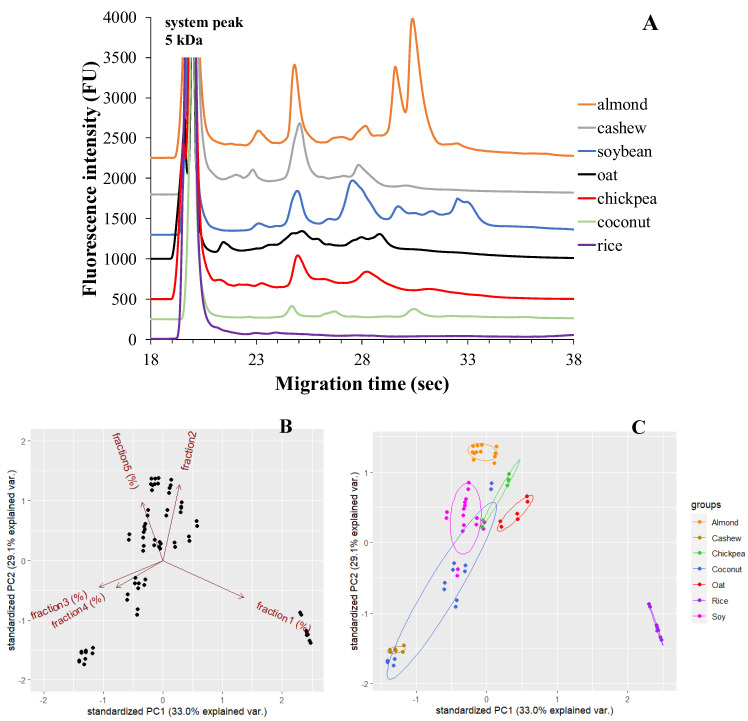
MGE protein profiles of seven different plant-based milk alternatives (**A**). The optimized experimental conditions are detailed in Materials and Methods. Principal component analysis (PCA) by % of total time-corrected area of protein fractions between different types of plant-based drinks. Loading plots (**B**) of principal component PC1 versus PC2; score plots (**C**) of the % of total time-corrected area of protein fractions for each plant-based drink from PC1 and PC2.

In Figure 1A, there are representative microchip gel electrophoretic protein profiles of seven different plant-based milk alternatives. Each plant-based drink exhibits a characteristic protein profile that is visualized as peaks (protein fraction) on the electropherogram. The number of protein fractions and their intensities vary among the different plant-based drinks.

The variability in protein content among the different types of plant-based drinks (i.e., oat, rice, chickpea, cashew, almond, coconut, soy) was described by two principal components (PCs).

In the PCA analysis, 2-dimensional data from 90 samples are projected onto the plane spanned by their two principal components (highlighted by the superimposed 95% confidence ellipses). The projection of the data points onto the PCA plot shows a clear separation between the rice (purple dots), oat (red dots), and almond (orange dots) drink samples and the others. The cumulative proportion of the first and second principal components explains nearly 62% of the total variability among products, 33.0% (PC1) and 29.1% (PC2), respectively. A high inter-product variability was observed for soy (pink dots) and coconut (blue dots) drinks (Figure 1C). The first and second fraction of protein content positively loaded PC1, while PC2 showed positive loadings for fraction 5. Fraction 3 and 4 were negligible on both PCs (Figure 1B).

#### 2.2.1. Protein Content of Cereal-Based Plant Drinks

##### Oat-Based Plant Drinks

Products from one brand with three different expiry dates were tested by duplicate measurements. The electrophoretic profile of the oat-based drink is shown in Figure 1**.** After the appearance of the system peak, fraction 1 with a molecular mass of ~9 kDa probably corresponds to an oat prolamin called avenin. This is followed by fraction 2 in the separation, which may be the ~24 kDa globulin (12S globulin) protein fraction, and finally fraction 3, probably also the globulin protein fraction, including 12S globulin with a molecular mass of ~54 kDa. The identification of fractions is based on previous studies [31,35].

Table 2 provides information on the values of three defined protein fractions in the oat drink. Among these fractions, fraction 2 contains approximately 52% and fraction 3 around 35% of the total protein content. According to Mäkinen [35] these findings suggest that globulin is the major protein fraction, constituting over 80% of protein abundance. On the other hand, avenin, a protein specific to oats, is present in a smaller percentage compared to the other fractions.

##### Rice-Based Plant Drinks

**Figure 2:**
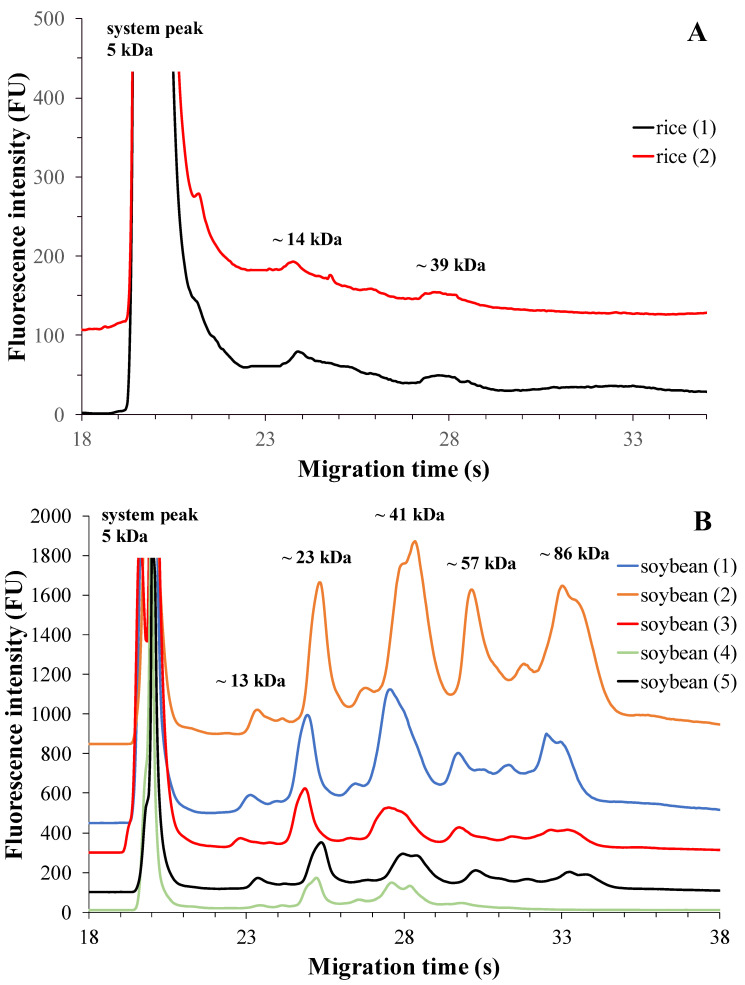
MGE protein profiles of rice-based plant drinks from two different brands (**A**) and soy-based plant drinks from five different brands (**B**). The optimized experimental conditions are detailed in Materials and Methods. Total protein concentrations applied in the chip well were roughly 0.5 µg/µL and 5 µg/µL, respectively.

Products from two brands with three different expiry dates were tested by duplicate measurements. The electrophoretic profile of the rice-based drinks is shown in Figure 2A. Using the specified measurement conditions, two protein fractions were separated in these rice drinks. The fractions were identified as reported by Wang et al. and Liu et al. More specifically, fraction 1 with ~14 kDa molecular weight was identified as the globulin fraction, while fraction 2 with ~39 kDa was identified as the albumin fraction [11,12]. These protein fractions may also contain respiratory allergens present in rice. Based on this knowledge, fraction 1 contains the profilin A (Ory s 12) allergen, and fraction 2 contains the rice allergen β-expansin (Ory s 1) [11]. Table 2 presents the protein content of rice drinks from two brands, specifically focusing on the two defined protein fractions. It is observed that the globulin protein fraction is present in the tested rice drinks in much higher proportions than the albumin fraction. Globulin accounts for approximately 80% of the total protein content, while albumin represents around 20%. These results align with the findings reported by Wang et al. [11].

#### 2.2.2. Protein Content of Legume-Based Plant Drinks

##### Chickpea-Based Plant Drinks

Products from one brand with three different expiry dates were tested by duplicate measurements. The electrophoretic profile of the chickpea-based drink is shown in Figure 1. Electropherograms presented four different protein fractions. Fraction 1 and fraction 2 usually had molecular weights of ~14 kDa and ~23 kDa, respectively. Based on a study by Grasso and co-workers, the globulin protein fraction can be assigned to these two peaks, with the monomeric form of 7S vicilin assigned to fraction 1, and the major protein in chickpeas, 11S legumin, assigned to fraction 2. These protein fractions are followed by fraction 3, glutelin, with ~44 kDa molecular weight. Then, convicilin with ~64 kDa appears as fraction 4 [19]. Concerning chickpea-based drinks, Table 3 displays the results of testing the same brand with three different expiry dates. Among the four protein fractions we analyzed, fraction 2 has the highest amount, accounting for approximately 60% of the protein composition. This fraction corresponds to 11S globulin, specifically legumin, which is known to be the major protein in chickpeas. The 7S vicilin fraction, in its monomeric form, represents the smallest fraction, comprising only about 2% of the protein content. Quantitative analysis indicates that the overall globulin content in chickpea beverages is more than 60%. Furthermore, chickpea drinks also contain the protein fractions glutelin (~27%) and convicilin (~12%) [19].

##### Soybean-Based Plant Drinks

The electrophoretic profiles of soy-based plant drinks from five different brands with three different expiry dates are shown in Figure 2B.

In addition to the system peak, five protein fractions were found during the separation. The electrophoretic profile clearly illustrates their migration, showing a sequence of fraction 1 (~13 kDa), fraction 2 (~23 kDa), fraction 3 (~41 kDa), fraction 4 (~57 kDa), and fraction 5 (~86 kDa). The first three fractions correspond to the 11S globulin fraction, while fraction 4 and fraction 5 correspond to the 7S globulin fraction [33].

Another study suggests that fraction 4 may also contain the immunodominant allergen Gly m Bd 30 K (P34). Fraction 2 may also contain basic subunits of glycine, while fraction 3 may contain acidic subunits of glycine [16]. The 7S globulin protein fraction consists of α’, α and β subunits of β-conglycine [16,17,33].

Soybean is a legume crop that, unlike chickpeas, has many allergens, but it also has a much broader protein palette and higher protein content [5,16]. As for soy-based drinks, Table 3 presents the results of testing different brands. The 11S globulin protein fraction (identified as fraction 1, fraction 2, and fraction 3) is the predominant protein component in soy drinks, constituting approximately 75–80% of the total protein content except in Brand 2 (Table 3). This fraction remains dominant across all brands. Fractions 4 and 5, which correspond to the 7S globulin fraction (specifically β-conglycinin), also exhibit dominant peaks, ranging from 20% to 25% of prevalence among all brands, except in Brand 2. The ratio of the 11S/7S globulin fractions ranged from 1.6 to 3.9, consistent with the values obtained in a study by Blazek and Caldwell, where a Bioanalyzer was also employed [33]. Analyzing the 11S and 7S globulin peaks separately, the smallest peak area was determined for fraction 1 and fraction 4. However, the fourth brand of soy drink showed a different distribution of protein fractions in terms of total protein content. In this case, fraction 3 was identified as the major protein component, comprising more than 50% of the total protein content. Nonetheless, the 11S/7S ratio in this brand was found to be approximately 2.5, which aligns with the findings of Blazek et al. [33].

Based on the Kruskal–Wallis test, there was no significant difference in the percentage of total corrected area of protein fractions between brands of soy-based plant drinks (fraction 1: *p* = 0.220; fraction 2: *p* = 0.439; fraction 3: *p* = 0.192; fraction 4: *p* = 0.060; fraction 5: *p* = 0.187).

#### 2.2.3. Protein Content of Nut-Based Plant Drinks

##### Cashew-Based Plant Drinks

Products from one brand with three different expiry dates were tested by duplicate measurements. The electrophoretic profile of the cashew-based plant drink is shown in Figure 1. Five different protein fractions were determined in the cashew-based drink, similar to Liu et al. [28]. From the obtained electropherograms, the following protein fractions can be identified, based on the previous study: fraction 1 with a molecular weight of ~10 kDa can be assigned to albumin, fraction 2 with a molecular weight of ~13 kDa can be assigned to the 2S albumin protein fraction (Ana o 3), fraction 3 with a molecular weight of ~24 kDa can be assigned to globulin, fraction 4 with a molecular weight of ~43 kDa can be assigned to the 11S globulin or legumin protein fraction (Ana o 2), and, finally, fraction 5 with a molecular weight of ~59 kDa can be assigned to the glutelin protein fraction. However, the last peak may also contain the vicilin-like Ana o 1 allergen [28,36]. Regarding cashew-based plant drinks, Table 4 presents the results of measurements conducted on beverages from one brand with three different expiry dates. A significant portion of the protein content in these cashew beverages, exceeding 65%, is attributed to fraction 3, that is, probably, the globulin protein fraction. Moreover, the 11S globulin or legumin protein fraction (fraction 4) exhibits a dominant peak, representing approximately 25% of the distribution fraction. Liu et al. also identified the globulin protein fraction as a major protein in their work [28]. The other fractions contribute to a minor part of the total protein content, accounting for about 10%. Cashew nut drinks consist of several protein components, including allergenic components.

##### Almond-Based Plant Drinks

**Figure 3:**
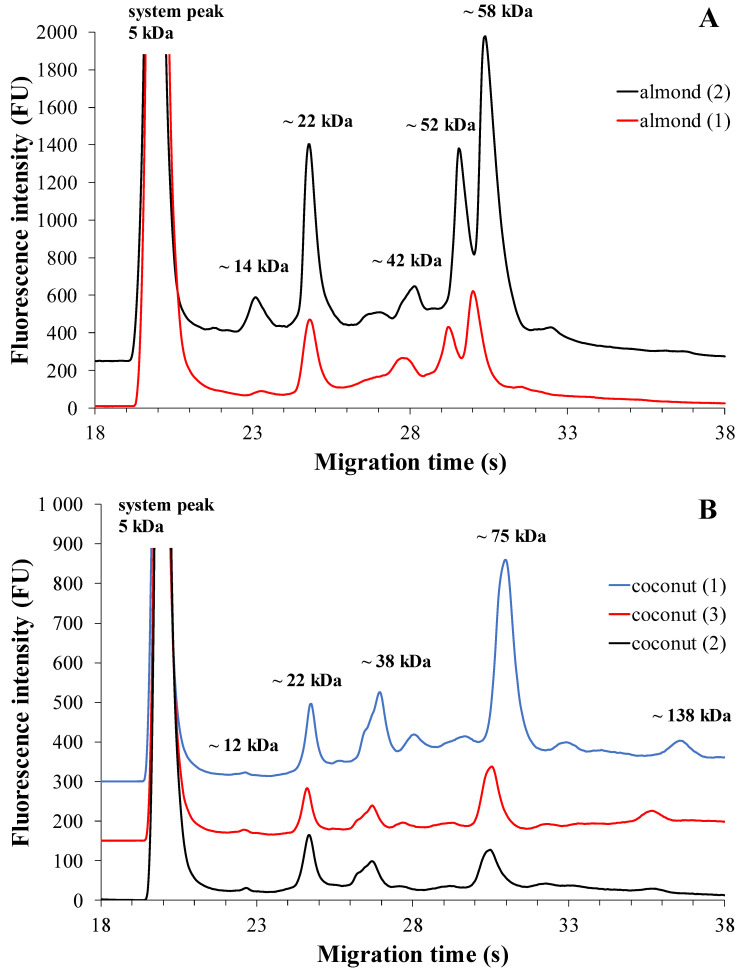
MGE protein profiles of almond-based plant drinks from two different brands (**A**) and coconut-based plant drinks from three different brands (**B**). The optimized experimental conditions are detailed in Materials and Methods. Total protein concentration applied in the chip well was roughly 3 µg/µL.

The electrophoretic profiles of almond-based plant drinks from two different brands with three different expiry dates show similar protein profiles (Figure 3A). Electropherograms showed five dominant fractions in addition to the system peak.

Based on previous studies, fraction 1 can be assigned to the nsLTP (Pru du 3) (~14 kDa), fraction 2 to the protein fraction of profilins, including Pru du 4 (~22 kDa), and fraction 3 to the 60S ribosomal protein (Pru du 5) (~42 kDa). The major protein fraction present in almonds is amandine (Pru du 6) (family of legumes) which can be identified as monomeric in our measurements. This protein fraction, Pru du 6, gives almonds their potential allergenicity. The two major proteins in amandine, the prunin-1 protein fraction (fraction 4) and the prunin-2 protein fraction (fraction 5), have a molecular mass of ~52 kDa and ~58 kDa, respectively. Considering reducing conditions, it is likely that fraction 1 contains the almond allergen Pru du 8, while fractions 2 and 3 also contain the acidic and basic subunits of prunin-1 and prunin-2 [21,22,37]. Concerning almond-based plant drinks, Table 4 displays the median values of drinks from two different brands, which show a similar protein composition. The results suggest that the major protein fractions in these plant-based beverages are profilins and prunin-2 protein (fraction 2 and 5). Additionally, the six major almond Pru du allergenic components (fraction 4 and fraction 5) are present in significant amounts, estimated to be approximately 50–55% and reported to be around 70% by De Angelis et al. [21]. Overall, almonds contain numerous protein fractions and, despite their allergenic nature, they are one of the most protein-rich plant-based beverages [6]. Based on the Mann–Whitney U-test results, there was no significant difference in the percentage of total corrected area of protein fractions between brands of almond-based plant drinks (fraction 1: *p* = 0.533; fraction 2: *p* = 0.267; fraction 3: *p* = 0.533; fraction 4: *p* = 1.000; fraction 5: *p* = 0.267).

##### Coconut-Based Plant Drinks

The electrophoretic profiles of coconut-based plant drinks from three different brands with three different expiry dates show a similarity in terms of protein profiles (Figure 3B). However, there are detectable differences in the intensity levels. The electropherogram reveals the presence of five protein fractions with fluorescence intensities.

Furthermore, in agreement with previous studies, the glutelin-1 protein fraction (fraction 1) was seen in the electropherogram with a molecular mass of ~12 kDa, prolamins (fraction 2) with a molecular mass of ~22 kDa, and albumin with a molecular mass of ~38 kDa. On the other hand, 11S globulins (fraction 4 and fraction 5), i.e., the monomeric and dimeric protein fractions of cocosin, could be identified by their molecular masses of ~75 kDa and ~138 kDa, respectively [22,23]. As for coconut-based drinks, Table 4 reveals the identification of several protein fractions. When testing three different brands, different protein component distributions were observed, with the highest proportion varying among brands. Brand 1 and 3 were found to contain the highest number of 11S globulin (cocosin) protein fractions, specifically fraction 4 and fraction 5, which is consistent with the findings reported by Patil et al. [23]. In coconut drinks from Brand 2, the albumin protein fraction (fraction 3) exhibited the highest % of TCA, and this protein fraction showed different values in all three brands. Among the minor proteins present, the proportion of glutelin-1 (fraction 1) ranged from 1% to 2%, while the prolamin fraction (fraction 2) accounted for 20% to 30%. Coconut-based drinks, in addition to being rich in energy and nutrition, contain multiple protein fractions, making them a significant part of a health-conscious diet [6].

Based on the Kruskal–Wallis test results, there was no significant difference in the percentage of total corrected area of protein fractions between brands of coconut-based plant drinks (fraction 1: *p* = 0.135; fraction 2: *p* = 0.546; fraction 3: *p* = 0.455; fraction 4: *p* = 0.546; fraction 5: *p* = 0.089).

## 3. Materials and Methods

### 3.1. Plant-Based Milk Alternative Samples

We selected plant-based milk alternatives (PBMAs) which were purchased in local grocery stores in Hungary, in the European Union, between 2019 and 2022, in carton packs that did not require refrigeration before opening, and were made from a single plant, salt, and drinking water. PBMAs that contained other additives such as sugars, flavorings, emulsifiers, oils, or other plant-based ingredients were excluded. Seven different PBMA varieties (five soy, three coconut, two rice, two almond, one oat, one chickpea and one cashew) were tested. The soy-, coconut-, rice-, and almond-based drinks were made by different brands and a total of fifteen drinks were measured. After randomization, which was performed before unpacking the PBMAs, the samples were incubated at 37 °C in a JULABO U3 circulating water bath (Julabo GmbH, Seelbach, Germany), shaken in their original packaging, and, after homogenization, poured into 10 mL centrifuge tubes and stored at −20 °C until analysis. For each product, duplicate analyses were performed on samples with three different expiry dates.

### 3.2. Microchip Gel Electrophoresis

The analysis of proteins in PBMAs was conducted using High Sensitivity Protein 250 (HSP 250) LabChip kits and an Agilent 2100 Bioanalyzer system equipped with a LIF (laser-induced fluorescence) detector (red laser diode, λ_max_ = 635 nm, excitation wavelength λ = 630 nm, and absorption wavelength λ = 680 nm). The HSP 250 LabChip kit provided the necessary microchips and reagents for the assays, including High Sensitivity Protein 250 Labeling Dye, DMSO, ethanolamine, Protein 250 standard labeling buffer (300 mM Tris/HCl, pH > 8.5), gel matrix (4.5% polydimethyl acrylamide-based linear polymer solution at pH 8), destaining solution, sample buffer, and ladder (containing seven proteins of known molecular weight and concentration). The denaturing solution containing SDS and dithiothreitol (DTT) was prepared by adding 3.5 µL 1 M DTT (Boehringer Mannheim GmbH, Mannheim, Germany) to 100 µL sample buffer. The original protocol was optimized for plant-based drink proteins to obtain a high efficiency of separation and sensitivity. In brief, fluorescently labelled proteins were prepared by combining 5 µL sample volume with 0.5 µL diluted fluorescent dye/DMSO solution (dye diluted 10-fold with water compared to the original protocol) and incubated for 10 min at room temperature (instead of 30 min incubation on ice as in the original protocol). Excess dye (i.e., unbound dye) was quenched by the addition of 0.5 µL ethanolamine (creating a complex with the excess dye unbound to the proteins providing system peaks for the evaluation, and thus able to be considered as an internal standard) and incubated for 10 min at room temperature (instead of 30 min incubation on ice as in the original protocol). The labeled samples were diluted two or five times (depending on the nominal concentration of the plant-based drink) by the addition of deionized water (instead of 200-fold dilution as in the original protocol). Four μL of this diluted sample solution was mixed with 2 µL denaturing solution, incubated for 5 min at 100 °C, and centrifuged. From each sample, 6 µL was loaded into the channels filled hydrodynamically with a polydimethylacrylamide-based linear polymer separation gel (pH 8). The destaining solution was loaded into the corresponding well. Samples were injected with 1000 V for 80 s (injection volume was approximately 40 pL) and the separation was continued toward the anode at 1000 V for 60 s at 30 °C.

Two parallel measurements were performed for each sample, and the protein fractions were manually integrated. The quantitative and qualitative evaluation of the fractions were carried out using the Agilent 2100 Expert software. The molecular masses of the protein components were determined using calibration curves [34]. From the time-corrected area under the curve (TCA) of the components, relative proportions were calculated and expressed as % of total TCA.

### 3.3. Structure of the Dataset

The dataset of food allergens was collected from the databases of the World Health Organization and International Union of Immunological Societies (WHO/IUIS) Allergen Nomenclature Sub-Committee (http://www.allergen.org, accessed on 28 August 2023). Protein sequences and functional information were obtained from the UniProt Database (http://www.uniprot.org, accessed on 28 August 2023).

### 3.4. Statistical Analysis

Migration time, molecular weight, and percentage of time-corrected area values were processed using the SPSS version 28.0 statistics software (IBM, New York, NY, USA). First, the normality of data distribution was checked and rejected. Variables were expressed as median (interquartile range). The Wilcoxon signed-rank test was used to determine any differences in total protein contents per 100 mL among measured and nominal concentrations. Meanwhile, the Kruskal–Wallis non-parametric one-way ANOVA for independent samples with multiple pairwise comparisons and Mann–Whitney signed-rank test were used to determine differences in total protein contents per 100 mL among various plant-based drink types and different brands. *p*-values less than 0.05 were considered to be significant. To explore the variability of protein contents among plant-based drink types, principal component Analysis (PCA) with varimax rotation was conducted using R Studio program (Version: 2023.06.0 + 421).

## 4. Conclusions

The microchip gel electrophoretic method for protein determination in PBMAs presented in this paper is a pioneer in analytical chemistry. According to the obtained results, the microchip gel electrophoretic method is suitable for the determination of protein fractions and total protein concentrations in plant-based drinks, providing valuable information about their protein profiles. In addition to the fluorescent labeling of samples, no further sample preparation step is required for the measurement, resulting in low sample and reagent (microliter) consumption. Overall, a complete assay of ten samples (including sample preparation and protein quantities) can be performed in less than one hour. A laser-induced fluorescence detector was used to detect the proteins, which is a sensitive detection method in terms of labeling reaction at a total protein content of 1 ng/µL. The method can minimize or eliminate matrix effects during the measurement. In addition to its many advantages, microchip gel electrophoresis also offers the possibility to perform routine laboratory tests rapidly. By considering both the variability of fractions’ position and the different numbers of fractions, this visual identification approach proved successful. The method effectively quantified the relative amount of protein fractions in the samples, allowing for differentiation based on protein profile. This information can be valuable for individuals with dietary restrictions or those seeking alternative protein sources. This research can help consumers make informed choices and promote the development and improvement of PBMAs. Moreover, the analysis also reveals that different plant-based beverages exhibit variations in their protein content. This diversity allows for the possibility of combining different plant-based drinks to achieve a more balanced amino acid profile and enhance the overall protein content. This combination of non-allergenic plant beverages can serve as a potential substitute for animal-based milks, providing adequate protein profiles. This finding supports the growing popularity and acceptance of plant-based diets and provides individuals with more options for meeting their protein needs. In our future study, we will investigate how PBMAs enriched with additives modify protein and nitrogen content.

## Figures and Tables

**Table 1:** Median (interquartile range) values of total protein content in different plant-based drinks compared to the nominal total protein concentrations on the package.

Product		Measured TP	Nominal TP
		g/100 mL	
**Oat (*n* = 6)**		2.0 (1.9–3.2)	1.4
**Rice (*n* = 12)**		0.35 (0.25–0.4)	0.2
**Chickpea (*n* = 6)**		2.6 (2.2–2.8)	2.0
Soy * (n = 30)	**Brand 1 (*n* = 6)**	4.6 (3.95–5.0)	3.6
	**Brand 2 (*n* = 6)**	4.7 (4.7–5.1)	4.1
	**Brand 3 (*n* = 6)**	4.3 (3.85–4.35)	3.5
	**Brand 4 (*n* = 6)**	3.8 (3.5–4.05)	2.6
	**Brand 5 (*n* = 6)**	4.2 (4.1–4.6)	3.2
**Coconut (*n* = 6)**		2.0 (1.7–2.8)	1.7
**Cashew (*n* = 18)**		1.8 (1.75–1.85)	1.0
**Almond (*n* = 6)**		2.6 (1.9–2.8)	1.1

* Different brands have different total protein concentrations on the label. TP: total protein content.

**Table 2:** MGE separation results for protein fractions in cereal-based plant drinks (molecular weight, migration time, % of total time corrected area) from the same brand (oat) and two different brands (rice), with three different expiry dates. The values are presented as medians (interquartile range) and based on duplicate measurements.

Cereal-Based Plant Drinks		MW (kDa)	Migration Time (s)	% of Total TCA
**Oat (*n* = 6)**	**fraction 1**	9.1 (8.8–9.2)	21.4 (21.4–21.4)	12.7 (10.9–13.0)
	**fraction 2**	24.0 (23.6–25.1)	25.2 (24.9–25.3)	51.8 (50.5–54.8)
	**fraction 3**	53.6 (51.1–53.7)	28.8 (28.8–29.2)	35.0 (32.3–38.3)
**Rice (*n* = 12)**	**Brand 1 (*n* = 6)**			
	**fraction 1**	14.4 (14.1–15.1)	23.9 (23.7–24.2)	83.2 (82.9–84.7)
	**fraction 2**	38.3 (35.7–40.7)	27.7 (27.1–28.0)	16.9 (15.3–17.1)
	**Brand 2 (*n* = 6)**			
	**fraction 1**	14.6 (14.1–14.8)	24.0 (23.9–24.1)	75.6 (75.0–76.2)
	**fraction 2**	39.0 (36.3–41.0)	27.8 (27.4–28.0)	24.4 (21.3–25.3)

**Table 3:** MGE separation results for protein fractions of legume-based plant drinks (molecular weight, migration time, % of total time-corrected area) from the same brand (chickpea) and five different brands (soy), with three different expiry dates. The values are presented as medians (interquartile range) and based on duplicate measurements.

Legume-Based Plant Drink		MW (kDa)	Migration Time (s)	% of Total TCA
**Chickpeas (*n* = 6)**	**fraction 1**	13.5 (12.6–13.5)	23.1 (22.7–23.1)	2.3 (2.1–2.3)
	**fraction 2**	22.7 (22.4–22.7)	24.7 (24.6–24.7)	63.9 (57.1–64.5)
	**fraction 3**	43.7 (43.6–44.5)	27.5 (27.4–27.6)	27.0 (24.2–29.9)
	**fraction 4**	63.6 (62.3–64.3)	29.8 (29.6–30.1)	12.3 (9.1–13.4)
**Soy (*n* = 30)**	**Brand 1 (*n* = 6)**			
	**fraction 1**	13.5 (13.4–13.8)	23.1 (23.1–23.3)	5.2 (4.1–5.8)
	**fraction 2**	23.0 (23.0–23.9)	24.9 (24.9–25.2)	29.8 (27.4–31.2)
	**fraction 3**	42.4 (41.9–42.7)	27.6 (27.5–27.8)	43.0 (37.0–49.4)
	**fraction 4**	58.8 (57.7–59.5)	29.7 (29.6–30.1)	6.7 (5.9–6.8)
	**fraction 5**	91.3 (88.8–93.2)	32.5 (32.3–33.0)	22.5 (12.7–23.3)
	**Brand 2 (*n* = 6)**			
	**fraction 1**	13.8 (13.7–14.3)	23.4 (23.3–23.5)	2.1 (2.0–2.6)
	**fraction 2**	24.1 (24.0–24.5)	25.2 (25.1–25.3)	20.7 (20.1–27.5)
	**fraction 3**	45.7 (45.4–45.7)	28.0 (28.0–28.2)	36.6 (32.2–38.3)
	**fraction 4**	59.3 (58.6–59.7)	29.9 (29.8–30.0)	12.6 (11.9–13.2)
	**fraction 5**	91.4 (90.0–92.5)	32.7 (32.6–32.9)	24.9 (24.3–26.5)
	**Brand 3 (*n* = 6)**			
	**fraction 1**	13.0 (12.8–13.4)	23.2 (23.0–23.2)	4.4 (4.2–5.8)
	**fraction 2**	21.0 (20.6–22.4)	25.0 (24.9–25.0)	33.6 (33.3–35.3)
	**fraction 3**	37.1 (36.2–39.2)	27.5 (27.3–27.5)	37.7 (36.5–38.6)
	**fraction 4**	52.4 (51.5–55.1)	29.7 (29.5–29.7)	8.4 (6.7–8.5)
	**fraction 5**	84.1 (81.4–89.2)	32.9 (32.7–33.0)	16.3 (13.7–17.5)
	**Brand 4 (*n* = 6)**			
	**fraction 1**	13.1 (13.0–13.7)	23.2 (23.2–23.3)	7.8 (5.1–8.0)
	**fraction 2**	19.9 (19.7–22.5)	25.0 (24.8–25.1)	26.5 (17.5–32.6)
	**fraction 3**	38.7 (37.0–40.8)	27.4 (27.3–27.5)	51.6 (48.3–53.4)
	**fraction 4**	51.0 (51.0–55.7)	29.4 (29.2–29.6)	6.7 (5.1–7.5)
	**fraction 5**	73.1 (73.1–74.3)	31.7 (31.3–31.8)	13.6 (6.9–18.7)
	**Brand 5 (*n* = 6)**			
	**fraction 1**	12.4 (12.4–12.9)	22.8 (22.7–23.1)	5.0 (5.0–5.3)
	**fraction 2**	20.6 (20.6–21.9)	24.8 (24.8–25.1)	32.1 (31.9–39.3)
	**fraction 3**	36.6 (36.6–38.3)	27.4 (27.4–27.7)	38.2 (36.5–38.5)
	**fraction 4**	52.4 (52.2–54.5)	29.7 (29.7–30.0)	8.2 (8.0–9.2)
	**fraction 5**	78.6 (75.2–82.6)	32.7 (32.4–33.0)	13.6 (8.0–17.0)

**Table 4:** MGE separation results for protein fractions of nut-based plant drinks (molecular weight, migration time, % of total time corrected area) from the same brand (cashew), two different brands (almond), or three different brands (coconut), with three different expiry dates. The values are presented as medians (interquartile range) and based on duplicate measurements.

Nut-Based Plant Drinks		MW (kDa)	Migration Time (s)	% of Total TCA
**Cashew (*n* = 6)**	**fraction 1**	10.2 (9.3–10.4)	21.9 (21.6–22.0)	2.4 (1.4–2.5)
	**fraction 2**	12.5 (12.5–12.6)	22.8 (22.7–22.8)	1.9 (1.9–4.1)
	**fraction 3**	23.7 (23.4–23.8)	25.0 (25.0–25.1)	68.9 (66.6–69.3)
	**fraction 4**	42.9 (42.6–43.6)	27.9 (27.9–27.9)	24.0 (22.6–24.2)
	**fraction 5**	59.1 (59.0–60.3)	30.1 (30.1–30.1)	4.7 (3.5–4.7)
**Almond (*n* = 12)**	**Brand 1 (*n* = 6)**			
	**fraction 1**	14.0 (13.9–14.2)	23.4 (23.3–23.4)	1.9 (1.8–2.3)
	**fraction 2**	21.8 (21.7–22.4)	24.9 (24.8–25.0)	31.3 (27.0–33.9)
	**fraction 3**	41.6 (41.2–42.8)	27.8 (27.7–28.0)	17.5 (15.9–17.9)
	**fraction 4**	52.7 (52.2–54.3)	29.3 (29.2–29.4)	12.4 (12.0–13.1)
	**fraction 5**	58.3 (58.0–61.9)	30.0 (30.0–30.2)	38.1 (34.9–40.7)
	**Brand 2 (*n* = 6)**			
	**fraction 1**	13.8 (13.8–13.9)	23.2 (23.2–23.3)	4.2 (3.1–4.4)
	**fraction 2**	21.8 (21.4–21.8)	24.7 (24.7–24.8)	27.6 (23.9–29.4)
	**fraction 3**	42.2 (41.9–42.4)	27.8 (27.6–27.8)	14.3 (13.8–14.5)
	**fraction 4**	52.1 (52.0–53.6)	29.1 (29.1–29.2)	14.3 (12.9–15.3)
	**fraction 5**	57.7 (57.4–59.4)	29.9 (29.9–29.9)	41.2 (37.9–45.6)
**Coconut (*n* = 18)**	**Brand 1 (*n* = 6)**			
	**fraction 1**	12.2 (12.2–12.3)	22.6 (22.5–22.6)	1.5 (0.5–2.5)
	**fraction 2**	21.5 (21.4–21.6)	24.6 (24.6–4.8)	21.5 (20.4–23.1)
	**fraction 3**	34.5 (34.2–34.8)	26.7 (26.6–27.0)	23.9 (21.3–24.5)
	**fraction 4**	75.4 (73.4–78.2)	30.7 (30.7–30.8)	48.3 (45.0–49.7)
	**fraction 5**	136.5 (128.2–143.0)	35.8 (35.7–35.8)	5.0 (4.5–5.1)
	**Brand 2 (*n* = 6)**			
	**fraction 1**	12.1 (11.7–12.3)	22.6 (22.5–22.6)	1.9 (1.0–2.2)
	**fraction 2**	21.2 (19.5–22.6)	24.7 (24.7–24.8)	30.1 (24.3–34.3)
	**fraction 3**	34.7 (30.9–38.2)	26.9 (26.6–27.2)	36,1 (33.3–43.2)
	**fraction 4**	75.7 (57.4–74.2)	31.0 (30.4–31.7)	29.8 (18.5–36.1)
	**fraction 5**	135.5 (127.0–139.9)	35.6 (35.6–35.7)	2.1 (1.5–3.6)
	**Brand 3 (*n* = 6)**			
	**fraction 1**	12.2 (12.2–12.3)	22.6 (22.6–22.6)	1.6 (1.0–1.7)
	**fraction 2**	22.5 (22.4–22.7)	24.6 (24.6–24.7)	23.2 (15.3–24.7)
	**fraction 3**	38.7 (38.7–38.9)	26.7 (26.7–26.7)	19.0 (14.5–22.6)
	**fraction 4**	75.7 (75.5–76.1)	30.5 (30.5–30.6)	44.2 (38.1–60.3)
	**fraction 5**	138.2 (138.0–139.0)	35.7 (35.7–35.8)	11.9 (8.9–12.9)

## Data Availability

Data is contained within the article.

## Sample Availability

Not available.

## Abbreviations

PBMA, plant-based milk alternatives; SDS-PAGE, sodium dodecyl sulfate–polyacrylamide gel electrophoresis; MGE, microchip gel electrophoresis; TCA, time-corrected area; PCA, principal component analysis; TP, total protein.
